# Teaching points in anesthetic management for transcatheter pulmonary valve replacement: a case series

**DOI:** 10.3389/fcvm.2026.1866846

**Published:** 2026-08-25

**Authors:** Meng Zhang, Siyuan Song, Jing Zhuang, Tong Wang, Qiang Lyu, Qian Lei

**Affiliations:** 1 Department of Anesthesiology, Sichuan Provincial People’s Hospital, University of Electronic Science and Technology of China, Chengdu, Sichuan, China; 2 Department of Anesthesiology, The Affiliated Hospital of Southwest Medical University, Luzhou, Sichuan, China; 3 Department of Neuroscience, Baylor College of Medicine, Houston, TX, United States; 4 Department of Anesthesiology, Montefiore Medical Center, Albert Einstein College of Medicine, Bronx, NY, United States; 5 Department of Biology, Duke University, Durham, NC, United States

**Keywords:** anesthesia, transcatheter pulmonary valve replacement, congenital heart disease, right ventricular dysfunction, pulmonary hypertension, pulmonary regurgitation, right ventricular outflow tract obstruction, perioperative management

## Abstract

**Background:**

Transcatheter pulmonary valve replacement (TPVR) is an established minimally invasive treatment for right ventricular outflow tract dysfunction after repair of congenital heart diseases. However, anesthetic management remains challenging because of complex right-sided pathophysiology and variable hemodynamic burden.

**Case:**

We retrospectively reviewed three representative single-center TPVR cases and summarized perioperative anesthetic management and early outcomes. The patients reflected distinct clinical patterns: severe RVOT obstruction with marked right ventricular pressure overload, severe pulmonary regurgitation with right ventricular volume overload, and complex adult congenital heart disease with pulmonary hypertension, atrial fibrillation, and shunt physiology. Despite these differences, anesthetic priorities were similar, including right ventricular protection, control of pulmonary vascular load, stable induction, lung-protective ventilation, close rhythm and hemodynamic monitoring, and preparation for critical procedural events.

**Conclusions:**

All three patients underwent TPVR successfully with stable early postoperative recovery, highlighting the importance of individualized but goal-directed anesthetic strategies.

## 1 Introduction

Transcatheter pulmonary valve replacement (TPVR) has become an important minimally invasive treatment for pulmonary valve dysfunction after surgical repair of congenital heart diseases (1). Compared with repeat open-heart surgery, TPVR is associated with less surgical trauma, faster recovery, and the possibility of repeat intervention when needed (2, 3). Because of these advantages, TPVR is being used increasingly in patients with right ventricular outflow tract (RVOT) dysfunction after previous repair.

Perioperative anesthetic management in this population remains challenging. Patients undergoing TPVR, particularly those with repaired congenital heart diseases and residual or recurrent RVOT or pulmonary valve dysfunction, may present with right ventricular enlargement or dysfunction, pulmonary hypertension, arrhythmias, prior sternotomy, calcified conduits, and limited hemodynamic reserve (4, 5). A clear understanding of the predominant hemodynamic abnormality is essential before the procedure, including whether the patient is primarily affected by RVOT obstruction with right ventricular pressure overload, severe pulmonary regurgitation with right ventricular volume overload, or mixed physiology (6, 7). Some patients have both right ventricular pressure (8, 9) as well as volume overload. Additional conditions such as atrial septal defect, atrial fibrillation, coronary artery disease, pulmonary hypertension, and advanced heart failure may further increase the risk of perioperative hemodynamic instability, arrhythmia, right ventricular decompensation, and hypoxemia (10, 11).

Anesthetic management for TPVR should therefore be individualized. The strategy should be based on the underlying pathophysiology and the key stages of the intervention. In this single-center retrospective case series, we describe three representative TPVR cases characterized by distinct right-sided hemodynamic patterns: severe RVOT obstruction with right ventricular pressure overload, severe pulmonary regurgitation with right ventricular volume overload, and complex mixed right-sided disease. We aimed to summarize practical perioperative anesthetic considerations across these different clinical scenarios.

## 2 Methods

This retrospective descriptive case series was conducted at a single center and included three patients who underwent TPVR. The cases were selected to represent distinct hemodynamic patterns encountered in clinical practice: severe RVOT obstruction with right ventricular pressure overload, severe pulmonary regurgitation with right ventricular volume overload, and complex adult congenital heart disease with mixed right-sided pathology.

Clinical data were retrospectively extracted from the medical records. The collected variables included demographic characteristics, presenting symptoms, cardiac history, preoperative imaging and catheterization findings, anesthetic agents, airway management, ventilator settings, invasive monitoring, vasoactive and inotropic support, intravenous fluid administration, ECMO support when applicable, intraoperative hemodynamic events, and early postoperative outcomes. Particular attention was given to lesion-specific anesthetic management during induction, catheter manipulation, balloon dilation, valve deployment, and emergence from anesthesia. Given the descriptive nature of the case series, no statistical comparisons were performed. The analysis focused on perioperative anesthetic management and practical teaching points across different right-sided hemodynamic patterns. The following section describes the clinical features, anesthetic management, and early postoperative course of each case. The clinical and anesthetic features of the three cases are summarized in Table 1.

**Table 1:** Clinical and anesthetic features of the three cases.

Variable	Case 1	Case 2	Case 3
Age/sex	18 years/male	35 years/male	69 years/female
Main diagnosis	Post persistent truncus arteriosus repair severe RV-PA conduit stenosis	Severe pulmonary regurgitation after TOF repair	Congenital heart disease with pulmonary valve stenosis and severe regurgitation, severe tricuspid regurgitation, ASD, AF, PH, CAD
Dominant lesion	Severe RV-PA conduit stenosis obstruction	Severe pulmonary regurgitation	Mixed right-sided lesion with pulmonary hypertension and shunt physiology
Main pathophysiology	Marked right ventricular pressure overload	Right ventricular volume overload and dilatation	Right heart dysfunction, pulmonary hypertension, atrial fibrillation, and ASD shunt
Key preoperative findings	NT-proBNP 2,929 pg/mL; frequent PVCs; severe conduit calcification and stenosis; marked RV hypertrophy; severe TR	Severe PR; enlarged RV; RVEF 41.31%; estimated PASP 46 mmHg	NT-proBNP 1,519 pg/mL; AF; severe PR and PS; severe TR; ASD with bidirectional shunt; precapillary PH
Anesthetic support	General anesthesia; VA-ECMO support; temporary pacing backup	General anesthesia; no ECMO support	General anesthesia; no ECMO support
Intervention	Covered stent implantation + TPVR	TPVR with VenusP P28-25 valve	TPVR as first-stage intervention
Main anesthetic concern	Hemodynamic collapse, arrhythmia, severe RV pressure overload, RV-PA conduit rupture/dissection	Preload optimization and RV protection	Pulmonary hypertension, desaturation risk, rhythm control, careful fluid strategy
Early postoperative outcome	Stable recovery; ECMO removed at 48 h; BNP decreased to 259 pg/mL	Stable recovery; extubated immediately; RV size reduced	Stable recovery; extubated immediately after TPVR

## 3 Results

### 3.1 Case 1

An 18-year-old patient was admitted with chest pain and reduced exercise tolerance for more than 5 years. The patient had undergone repair of persistent truncus arteriosus with ventricular septal defect in infancy. A second operation was later performed for RVOT-main pulmonary artery obstruction. Follow-up showed progressive restenosis of the RVOT-main pulmonary artery conduit. Exercise tolerance gradually declined, and dyspnea became evident during daily activity.

Preoperative evaluation showed an NT-proBNP level of 2,929 pg/mL. Electrocardiography demonstrated sinus rhythm with frequent premature ventricular contractions. Echocardiography, pulmonary artery computed tomography, and cardiac magnetic resonance imaging showed severe calcification and marked stenosis of the RVOT-main pulmonary artery conduit, right heart enlargement, marked right ventricular hypertrophy with reduced systolic function, compression of the left ventricle, and severe tricuspid regurgitation. Right heart catheterization showed a right atrial pressure of 19/10/13 mmHg, a right ventricular pressure of 180/20/73 mmHg, and a pulmonary artery pressure distal to the stenosis of 15/5/8 mmHg.

The patient underwent covered stent implantation in the RVOT-main pulmonary artery followed by TPVR under general anesthesia. Norepinephrine was prepared before induction and initiated at approximately 0.03 μg/kg/min, with titration within a range of 0.03–0.08 μg/kg/min to maintain adequate systemic arterial pressure and right ventricular coronary perfusion. Epinephrine was prepared for rescue inotropic support but was not administered because no persistent hypotension or clinically significant deterioration in ventricular contractility occurred. Given the extreme right ventricular pressure overload, impaired right ventricular systolic function, and severe tricuspid regurgitation, anesthetic induction was performed cautiously, with particular attention to preserving systemic vascular resistance and right ventricular coronary perfusion. General anesthesia was induced with etomidate 0.2 mg/kg, sufentanil 0.3 μg/kg, and rocuronium 0.6 mg/kg, followed by endotracheal intubation. Anesthesia was maintained with sevoflurane at approximately 0.8–1.0 minimum alveolar concentration together with remifentanil at 0.05–0.15 μg/kg/min.

In addition to standard monitoring, continuous invasive arterial blood pressure and central venous pressure monitoring were used. Mechanical ventilation was performed in volume-controlled mode with a tidal volume of approximately 6–7 mL/kg predicted ideal body weight, a PEEP of 5 cmH₂O, and an initial FiO₂ of 0.5, subsequently adjusted according to oxygenation. Respiratory rate was adjusted to maintain an end-tidal carbon dioxide concentration of approximately 35–40 mmHg, while excessive airway pressure was avoided.

Because of the severe RV–PA conduit obstruction, extreme right ventricular pressure overload, impaired right ventricular systolic function, and the anticipated risk of acute circulatory collapse during balloon dilation and valve deployment, venoarterial ECMO support was established immediately after induction of general anesthesia. Initial ECMO blood flow was approximately 3.5 L/min, with a sweep gas flow of 2 L/min and an oxygenator FiO₂ of 1.0; support was subsequently adjusted according to systemic arterial pressure, oxygenation, and procedural requirements. Temporary pacing was established prior to percutaneous balloon dilation because of the frequent ventricular ectopy and the risk of bradyarrhythmia or conduction disturbance during guidewire manipulation and valve deployment.

Hemodynamics were closely monitored during balloon dilation, stent deployment, and valve implantation. No sustained malignant arrhythmia or refractory hypotension occurred. Following successful TPVR, the patient was transferred with ECMO and mechanical ventilatory support. Chest computed tomography was performed 24 h later to evaluate possible pulmonary reperfusion injury. During transport, ECMO and mechanical ventilation were maintained with continuous electrocardiographic, oxygen saturation, and invasive arterial pressure monitoring. No evidence of pulmonary reperfusion injury was identified. ECMO support was continued because of the pre-existing severe right ventricular systolic dysfunction and the ongoing need for circulatory support. Weaning readiness was assessed during reduced ECMO flow using the following targets: a mean arterial pressure above 65 mmHg, a pulse pressure above 10 mmHg at an ECMO flow of 2.0–2.5 L/min without escalation of vasoactive support, adequate oxygenation and ventilation, satisfactory end-organ perfusion, and echocardiographic evidence of right ventricular recovery, with TAPSE and right ventricular fractional area change improving toward the normal reference values of >17 mm and >35%, respectively. ECMO flow was gradually reduced while monitoring arterial pressure, oxygenation, rhythm, and ventricular function. The patient tolerated the weaning trial, and ECMO was discontinued 48 h after the procedure. Dyspnea improved, and BNP decreased to 259 pg/mL.

### 3.2 Case 2

A 35-year-old man was admitted because of exertional fatigue. At 14 years of age, he had undergone surgical repair of tetralogy of Fallot, including ventricular septal defect closure, resection of obstructive muscular bundles, and repair of pulmonary valve stenosis. During the week before admission, exertional symptoms markedly worsened.

Electrocardiography showed sinus rhythm, premature atrial contractions, and complete right bundle branch block. Echocardiography showed severe pulmonary regurgitation, increased forward pulmonary valve flow velocity (2.15 m/s), a pulmonary valve gradient of 18.49 mmHg, marked right ventricular enlargement, right atrial enlargement, mild tricuspid regurgitation, and an estimated pulmonary artery systolic pressure of 46 mmHg. Pulmonary artery computed tomography angiography showed postoperative change and right heart enlargement. Cardiac magnetic resonance imaging showed a right ventricular end-diastolic volume of 159 mL/m^2^ and reduced right ventricular systolic function, with a right ventricular ejection fraction of 41.31%.

The patient underwent TPVR under general anesthesia without ECMO support. The principal anesthetic concern was chronic right ventricular volume overload associated with severe pulmonary regurgitation and right ventricular dilatation. General anesthesia was induced with propofol 1.0–1.5 mg/kg, sufentanil 0.3 μg/kg, and rocuronium 0.6 mg/kg. Propofol was administered incrementally to minimize abrupt reductions in systemic vascular resistance and venous return. Anesthesia was maintained with sevoflurane at approximately 0.8–1.0 minimum alveolar concentration together with remifentanil at 0.05–0.10 μg/kg/min.

Mechanical ventilation was performed in volume-controlled mode with a tidal volume of approximately 6–8 mL/kg predicted ideal body weight, PEEP of 5 cmH₂O, and an FiO₂ of approximately 0.4–0.5. Respiratory rate was adjusted to maintain an end-tidal carbon dioxide concentration of approximately 35–40 mmHg. Hemodynamic management focused on maintaining adequate right ventricular preload while avoiding excessive volume administration. Approximately 700 mL of balanced crystalloid was administered during the procedure. A low-dose norepinephrine infusion at approximately 0.02–0.05 μg/kg/min was used transiently during induction and discontinued after stabilization of arterial pressure. No additional inotropic support or ECMO was required.

A VenusP P28-25 pulmonary valve was implanted; the valve assembly was delivered through the right femoral vein. No sustained arrhythmia or clinically significant hemodynamic instability occurred during catheter manipulation or valve deployment. Following confirmation of stable hemodynamics and adequate spontaneous ventilation, the tracheal tube was removed at the end of the procedure. Pulmonary artery pressure returned to the normal range. The mean systolic transvalvular gradient was approximately 10 mmHg. Only trace tricuspid regurgitation remained, and right ventricular size was reduced after the intervention.

### 3.3 Case 3

A 69-year-old woman was admitted with fatigue and dyspnea. Her diagnoses included congenital heart disease with pulmonary valve stenosis and severe pulmonary regurgitation, severe tricuspid regurgitation, atrial septal defect, atrial fibrillation, chronic heart failure, pulmonary hypertension, and coronary artery disease.

NT-proBNP was 1,519 pg/mL. Electrocardiography showed atrial fibrillation and complete right bundle branch block. Echocardiography and pulmonary artery computed tomography showed a secundum atrial septal defect with bidirectional atrial shunting, pulmonary valve stenosis with severe regurgitation, pulmonary artery dilatation, elevated right ventricular systolic pressure, increased right atrial pressure, a dysplastic tricuspid valve with severe regurgitation, right ventricular hypertrophy, right atrial enlargement, mild mitral stenosis with mild-to-moderate regurgitation, left atrial enlargement, mild left ventricular hypertrophy, mild aortic regurgitation, and a small pericardial effusion. Coronary angiography showed mild-to-moderate coronary artery disease. Right heart catheterization confirmed precapillary pulmonary hypertension. Chest computed tomography also showed a bulla in the right middle lobe and mild inflammatory changes in both lungs.

After multidisciplinary discussion, a staged strategy was adopted. The RVOT lesion was treated first, and closure of the atrial septal defect was planned for a later stage after improvement in hemodynamics and cardiac function. The patient underwent TPVR under general anesthesia without ECMO support. Given the presence of precapillary pulmonary hypertension, severe tricuspid regurgitation, atrial fibrillation, bidirectional atrial shunting, and chronic right-sided heart failure, the principal anesthetic goals were preservation of systemic arterial pressure, avoidance of increases in pulmonary vascular resistance, maintenance of adequate right ventricular preload, and prevention of hypoxemia, hypercapnia, and acidosis.

General anesthesia was induced with etomidate 0.2 mg/kg, sufentanil 0.2–0.3 μg/kg, and rocuronium 0.6 mg/kg, followed by endotracheal intubation. Anesthesia was maintained with sevoflurane at approximately 0.6–0.8 minimum alveolar concentration together with remifentanil at 0.05–0.10 μg/kg/min.

Mechanical ventilation was performed with a tidal volume of approximately 6 mL/kg predicted body weight, PEEP of 4–5 cmH₂O, and an FiO₂ of 0.5. Respiratory rate was adjusted to maintain an end-tidal carbon dioxide concentration of approximately 35–38 mmHg, and excessive airway pressure was avoided.

Fluid administration was conservative because of severe tricuspid regurgitation and chronic right-sided volume overload. Approximately 500 mL of balanced crystalloid was administered during the procedure. Key anesthetic considerations during TPVR are summarized in Table 2. Norepinephrine was infused at approximately 0.03–0.06 μg/kg/min when necessary to maintain systemic arterial pressure. No ECMO support was required.

**Table 2:** Key anesthetic considerations during TPVR.

Stage	Key anesthetic considerations
Preoperative assessment	Review echocardiography, CT/MRI, ECG, and catheterization findings; identify the dominant lesion and main hemodynamic risk; prepare vasoactive drugs, temporary pacing, and rescue support if needed
Induction	Use a cardio-stable drug such as etomidate; avoid sudden vasodilation
Ventilation	Maintain oxygenation and normocapnia; avoid high airway pressure and factors that increase pulmonary vascular resistance
Catheter manipulation	Monitor closely for vagal response, bradycardia, and ventricular ectopy during guidewire passage
Balloon dilation	Provide oxygenation with 100% O₂ for 3 min prior to balloon dilation and be prepared for abrupt desaturation and hypotension due to transient interruption of pulmonary blood flow.
Valve deployment	Anticipate rapid changes in right ventricular loading conditions and systemic hemodynamics
Hemodynamic support	Individualized fluid administration, inotropes, and vasopressors according to the dominant lesion and right ventricular function
Rhythm management	Prepare for atrial or ventricular arrhythmias; consider pacing backup in high-risk patients
Recovery	Ensure stable hemodynamics, adequate analgesia, and continued right ventricular protection, rule out acute pulmonary perfusion injury by CT scan in severe RV-PA stenosis prior to weaning ECMO and ventilatory support, extubate trachea according to the standard extubation criteria

No sustained arrhythmia, severe hypotension, or significant oxygen desaturation occurred during valve deployment. After restoration of spontaneous ventilation and confirmation of stable oxygenation and hemodynamics, the tracheal tube was removed at the end of the procedure. Postoperative vital signs remained stable.

## 4 Summary of perioperative findings

The three patients had different dominant lesions and hemodynamic patterns. Case 1 was characterized by severe RV–PA conduit obstruction and extreme right ventricular pressure overload. Case 2 was dominated by severe pulmonary regurgitation and right ventricular volume overload. Case 3 showed mixed right-sided disease with pulmonary hypertension, atrial fibrillation, and shunt physiology. All three cases required individualized anesthetic planning based on preoperative hemodynamic assessment. In all patients, the key principles included preservation of right ventricular function, avoidance of factors that increase pulmonary vascular resistance, close monitoring of rhythm and hemodynamics, and careful management during critical procedural steps such as balloon dilation and valve deployment.

## 5 Discussion

This case series illustrates the heterogeneity of patients undergoing TPVR and emphasizes that anesthetic management should be determined by the dominant pathophysiologic abnormality rather than by the procedure itself. Although all three patients underwent TPVR, their perioperative risks were clearly different. In the patient with severe RV–PA conduit obstruction, the main concerns were extreme right ventricular pressure overload, conduit rupture or dissection, and the potential for abrupt hemodynamic deterioration. In the patient with severe pulmonary regurgitation, chronic right ventricular volume overload and progressive chamber dilatation were more prominent. In the most complex patient, mixed valvular disease, pulmonary hypertension, atrial fibrillation, and shunt physiology created a broader and more unstable hemodynamic profile. These differences indicate that TPVR should not be regarded as a uniform anesthetic condition.

Across these different clinical settings, preservation of right ventricular function remained the central objective (12, 13). This requires avoidance of factors that increase pulmonary vascular resistance, including hypoxemia, hypercapnia, acidosis, agitation, severe pain, and excessive airway pressure. Maintenance of adequate systemic pressure is essential, as right ventricular coronary perfusion may be particularly vulnerable in patients with advanced right-sided dysfunction. In the most severe case in this series, ECMO and temporary pacing were established before the highest-risk interventional steps because of severe RV pressure overload, impaired RV function, frequent ventricular ectopy, and the anticipated risk of abrupt circulatory deterioration during balloon dilation and valve deployment. This experience highlights the importance of defining rescue strategies before intervention, including immediate availability of vasoactive and inotropic support, pacing, and mechanical circulatory support in selected high-risk patients (14).

Another important observation from these cases is that preoperative evaluation should be organized around functional and hemodynamic assessment. The key issue is not only the anatomical diagnosis, but also whether the dominant process is pressure overload, volume overload, pulmonary hypertension, or mixed disease. Particular attention should be given to right ventricular size and systolic performance, tricuspid regurgitation, pulmonary artery pressure, the severity of conduit obstruction or pulmonary regurgitation, coronary artery disease, and rhythm abnormalities. Integration of echocardiographic findings with computed tomography or cardiac magnetic resonance imaging, electrocardiography, and catheterization data provides a more complete basis for perioperative risk stratification and anesthetic planning.

The intraoperative course of TPVR also demands careful anticipation of procedure-specific hemodynamic changes (13, 15). Induction should be gradual and hemodynamically stable, particularly in patients with impaired right ventricular reserve or pulmonary hypertension. Anesthetic agents should be selected and titrated according to the underlying hemodynamic profile to minimize myocardial depression, systemic vasodilation, and abrupt reductions in preload. In Cases 1 and 3, etomidate was selected to support hemodynamic stability, whereas propofol was administered cautiously in Case 2. Ventilation was adjusted to maintain oxygenation and normocapnia while avoiding excessive airway pressure. Fluid and vasoactive therapy were individualized according to right ventricular loading conditions and systemic arterial pressure. Rhythm disturbances require particular vigilance, as guidewire manipulation, balloon dilation, and valve deployment may precipitate clinically significant arrhythmias.

Several critical moments during TPVR deserve special attention. Before balloon inflation, the anesthesiology team should ventilate the patient with 100% O₂ for 3 min, optimize systemic arterial pressure and preload, ensure immediate availability of vasoactive and inotropic support, and verify rhythm monitoring and pacing backup in high-risk patients. Communication with the interventional team is essential so that balloon inflation and valve deployment can be anticipated and hemodynamic support adjusted promptly. Balloon dilation may transiently interrupt pulmonary blood flow and result in abrupt hypotension, desaturation, or arrhythmia, particularly in patients with limited right ventricular reserve (16, 17). Valve deployment may also rapidly alter right ventricular loading conditions, especially after relief of severe obstruction. Therefore, close hemodynamic and rhythm monitoring should be maintained throughout this transition, with prompt adjustment of vasoactive support, ventilation, and fluid administration when needed.

Several limitations should be acknowledged. This report describes only three patients from a single center, and the analysis is descriptive in nature. Long-term postoperative follow-up was not the focus of the present study. Even so, the cases represent several clinically relevant patterns encountered during TPVR and may offer practical insight into perioperative anesthetic management in this setting.

## 6 Conclusions

Anesthetic management for TPVR should be individualized according to the dominant lesion and the underlying hemodynamic pattern. Across different clinical scenarios, right ventricular protection remains the central principle. Careful preoperative assessment, tailored induction and ventilation, close hemodynamic and rhythm monitoring, and anticipation of critical procedural events are essential for safe perioperative management. This case series provides practical teaching points for anesthesiologists involved in TPVR and other complex structural heart interventions.

## Data Availability

The raw data supporting the conclusions of this article will be made available by the authors, without undue reservation.
